# Investigation of green synchronous spectrofluorimetric approach for facile sensitive estimation of two co-administered anti-cancer drugs; curcumin and doxorubicin in their laboratory-prepared mixtures, human plasma, and urine

**DOI:** 10.1186/s13065-024-01272-6

**Published:** 2024-09-09

**Authors:** Diaa Dagher, Heba Elmansi, Jenny Jeehan Nasr, Nahed El-Enany

**Affiliations:** 1 Department of Pharmaceutical Analytical Chemistry, Faculty of Pharmacy, Mansoura University, Mansoura, 35516 Egypt; 2 Department of Pharmaceutical Analytical Chemistry, Faculty of Pharmacy, Mansoura National University, Gamasa, 7723730 Egypt; 3 Department of Pharmaceutical Chemistry, Faculty of Pharmacy, New Mansoura University, New Mansoura, 7723730 Egypt

**Keywords:** Curcumin, Doxorubicin, Synchronous spectrofluorimetric, Biological matrices, Greenness

## Abstract

Recently, phytochemicals play an important role in cancer management. Curcumin (CUR), a natural phytochemical, has been co-administered with widespread chemotherapeutic agents such as doxorubicin (DOX) due to its excellent antitumor activity and the ability to lower the adverse reactions and drug resistance cells associated with DOX use. The present study aims to determine DOX and CUR utilizing a label-free, selective, sensitive, and precise synchronous spectrofluorimetric method. The obvious overlap between the emission spectra of DOX and CUR prevents simultaneous estimation of both analytes by conventional spectrofluorimetry. To solve such a problem, synchronous spectrofluorimetric measurements were recorded at Δλ = 20 nm, utilizing ethanol as a diluting solvent. Curcumin was recorded at 442.5 nm, whereas DOX was estimated at 571.5 nm, each at the zero-crossing point of the other one. The developed method exhibited linearity over a concentration range of 0.04–0.40 μg/mL for CUR and 0.05–0.50 μg/mL for DOX, respectively. The values of limit of detection (LOD) were 0.009 and 0.012 µg/mL, while the values of limit of quantitation (LOQ) were 0.028 and 0.037 µg/mL for CUR and DOX, respectively. The adopted approach was carefully validated according to the guidelines of ICH Q_2_R_1_. The method was utilized to estimate CUR and DOX in laboratory-prepared mixtures and human biological matrices. It showed a high percentage of recoveries with minimal RSD values. Additionally, three different tools were utilized to evaluate the greenness of the proposed approach.

## Introduction

Chemotherapy with antitumor medications is crucial in the clinical treatment of cancer. However, these medications have serious side effects, and the cancer cells may develop drug resistance that could lower chemotherapy’s effectiveness or even stop it [1, 2]. Since some phytochemicals have such strong anticancer action, they could be used in combination with chemotherapeutic drugs in cancer treatment. Additionally, phytochemicals are safer and more favorable than chemotherapy which has unwanted side effects [3, 4].

**Fig. 1:**
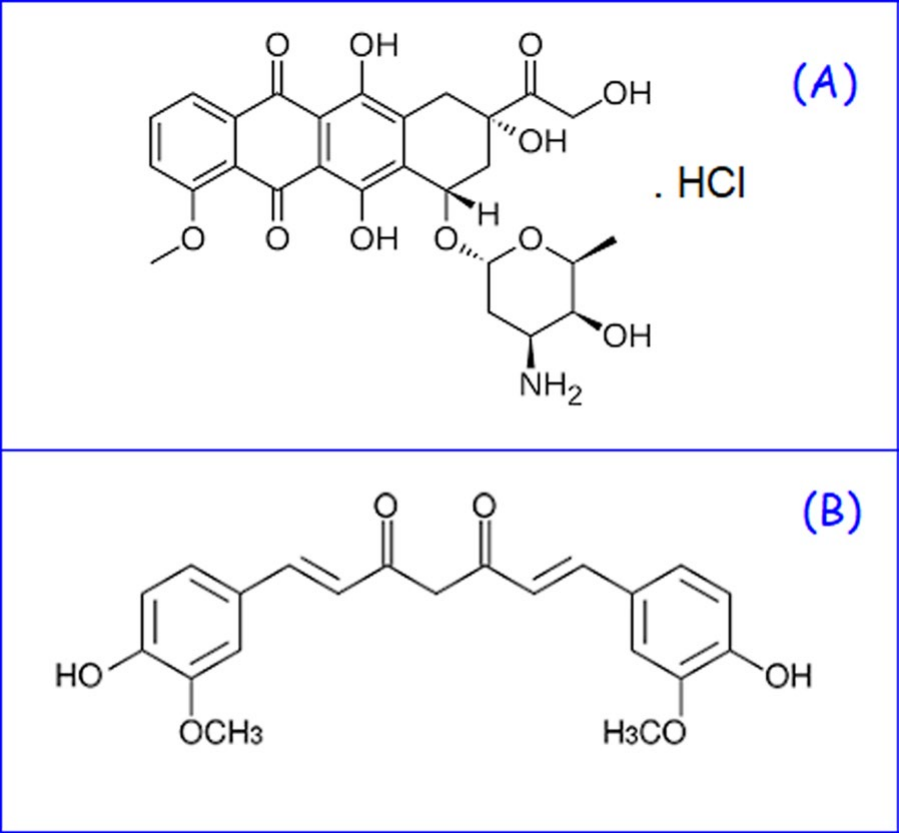
Structural formulae of DOX (**A**) and CUR (**B**)

Doxorubicin (DOX, Fig. 1A) hydrochloride, an anthracycline glycoside, is chemically named (8*S*,10*S*)-10-[(3-Amino-2,3,6-trideoxy-a-L-lyxo-hexopyranosyl)oxy]-6,8,11-trihydroxy-8-(hydroxyacetyl)-1-methoxy-7,8,9,10-tetrahydrotetracene-5,12-dione hydrochloride. DOX is frequently used to treat several malignancies, such as ovarian cancer, breast cancer, lung cancer, and malignant lymphoma [5, 6]. Its mechanism of action depends on the intercalation of DOX with the DNA double helix so that it inhibits transcription as well as replication of cancer cell DNA [7, 8]. Owing to the potential adverse reactions such as cardiotoxicity and subsequent congestive heart failure, the long-term clinical use of DOX is restricted [9, 10]. To avoid this problem, various strategies could be adopted such as using a natural product that decreases side effects and reduces cancer cells’ drug-resistance [11]. The literature survey of DOX revealed different assay methods such as spectrophotometric [12, 13], HPLC [12, 13], spectrofluorimetric [14–17], and electrochemical [18–20] methods.

Curcumin (CUR, Fig. 1B) is the polyphenolic bioactive component of *Curcuma longa* rhizome. CUR is chemically named (1E,6E)-1,7-bis(4-hydroxy-3-methoxyphenyl)hepta-1,6-diene-3,5-dione. CUR is commonly used as a food coloring agent due to its yellow color, preservative, and spice in cooking [21]. It has an impressive role in medicine by its antioxidant [22], antiproliferative [23], anti-inflammatory [24], immunomodulatory [25], antidiabetic [26], and antitumor [27] activity. Several methods for the assay of CUR have been published such as HPLC [28, 29], spectrophotometric [30, 31], spectrofluorimetric [32–35], and electrochemical [36–38] methods.

Curcumin, an important natural phytochemical, has been reported to be used in combination chemotherapy with doxorubicin [11, 39]. Curcumin increases the efficacy of DOX on cancer cells, decreases the side effects of DOX, and reverses the chemoresistance of DOX [39, 40].

Although both DOX and CUR were determined separately by various methods, only two HPLC approaches were reported for simultaneous estimation of both DOX and CUR [41, 42]. These HPLC methods [41, 42] had many disadvantages, such as complicated sample preparation, long chromatographic run times, and using environmentally hazardous solvents such as acetonitrile in the mobile phase. Up till now, no spectrofluorimetric approaches have been published for simultaneous estimation of both DOX and CUR. Considering that spectroscopic methods are the most widespread analytical techniques due to their simplicity and availability compared with chromatographic methods. The primary aim of the presented work is to validate a simple, rapid, reliable, ultra-sensitive, and selective spectrofluorimetric approach used for concurrent determination of both CUR and DOX in their laboratory-prepared mixtures, spiked human plasma and urine. The overlapped spectra produced from the direct measurements of the native fluorescence of both analytes were resolved by employing synchronous fluorescence spectroscopy (SFS) mode [43–47]. Three different tools were utilized to ensure the proposed approach’s greenness. The utilized three tools were the National Environmental Method Index (NEMI) [48], analytical ecoscale [49], and AGREE metric approach [50].

## Experimental

### Instrumentation

- A Cary Eclipse fluorescence spectrophotometer was utilized for all measurements. It was equipped with an 800-V xenon lamp. Furthermore, the selected slit width was 5 nm with a smoothing factor of 20, and the utilized Δλ was Δλ = 20 nm. Helma® fluorescence quartz cuvette cell was used for sample measurement.
- For the pH adjustment, a Consort pH meter (model NV P-901, Belgium) was utilized. A glass electrode was attached to the pH meter which contained Ag/AgCl reference electrode.
- For sonication, Sonic IV model-SS101H 230 (USA) was utilized.
- For biological fluids preparation, a vortex mixer (model IVM-300P, Taiwan) and a centrifuge (model 2-16P, Germany) were used.

### Reagents and materials

- Doxorubicin (99.85%) was obtained from Selleckchem (Houston, USA).
- Doxorubicin ‘Ebewe’ ® ampule 50 mg/25 mL batch # KV0787, EBEWE Pharma Ges.m.b.H.Nfg.KG A-4866 Unterach, Austria.
- Curcumin (99.0%) was purchased from EMITCO Pharmaceuticals (Alexandria, Egypt).
- Organic solvents (HPLC grade) such as acetonitrile, methanol, and ethanol were obtained from Sigma‐Aldrich, Germany.
- Sodium hydroxide, boric acid, acetic acid, phosphoric acid, β-cyclodextrin, carboxy methyl cellulose (CMC), sodium dodecyl sulfate (SDS), and Tween 80, were obtained from Sigma‐Aldrich, Germany.
- Human plasma samples utilized in this study were generously supplied by Mansoura University Hospital. These plasma samples were kept in the freezer till the analysis time.
- Fresh urine samples were donated by a 50-year-old drug-free, healthy male volunteer and were kept in the freezer till the analysis time.

### Standard solutions and buffer solutions

- Stock solutions (100 μg/mL) were prepared in 100 mL volumetric flasks by separately dissolving 10 mg of CUR in ethanol, and then completed to the mark with ethanol, while 10 mg of DOX was dissolved in distilled water then completed to the mark with distilled water. After that, the prepared solutions were diluted to obtain the working solutions with a concentration of 10 μg/mL for both DOX and CUR.
- Britton Robinson buffer (BRB) solution was prepared by mixing equimolar concentrations (0.4 M) of boric acid, acetic acid, and phosphoric acid. After that, the pH was adjusted utilizing 0.2 M sodium hydroxide, resulting in a series of solutions covering a pH range of 2–12.

### Construction of calibration curves procedures

The experimental procedure involved transferring specified volumes of each of DOX and CUR working solutions (10 μg/mL) into a set of 10 mL volumetric flasks, then diluted with ethanol to the mark and mixed well to obtain the final concentration range 0.05–0.50 μg/mL for DOX and 0.04–0.40 μg/mL for CUR. Afterward, SFS of the prepared solutions was measured at Δλ = 20 nm for each analyte. The synchronous fluorescence intensities were recorded for CUR and DOX against ethanol blank at 442.5 nm and 571.5 nm, respectively. All measurements were made at room temperature 25 ± 2 °C. After that, the calibration graphs were conducted by plotting the relative synchronous fluorescence intensity (RSFI) versus the corresponding final concentrations of each drug in μg/mL. Following that, the regression equations were generated using the data obtained from the calibration curves.

### Procedures for analysis of DOX/CUR in their laboratory-prepared mixtures

In a 100 mL volumetric flask, a stock solution (100 μg/mL) of DOX was prepared by dissolving 5 mL of Doxorubicin ‘Ebewe’ ® ampule 50 mg/25 mL into 50 mL distilled water, mixed well, and diluted to the mark with distilled water. After that, further dilution was made to prepare a 10 μg/mL working solution. Variable aliquots from DOX and CUR working solutions (10 μg/mL) were quantitatively transferred to a series of 10 mL volumetric flasks to prepare four laboratory-prepared mixtures with variable ratios of (2:1), (1:3), (1:2), and (4:3), respectively. After that, the flasks were completed with ethanol to the mark. The procedure was then followed as cited in “Construction of calibration curves procedures” section.

### Procedures for DOX/CUR analysis in spiked biological fluids

Two different sets of 15.0 mL centrifugation tubes were used to spike 1 mL of human plasma or urine separately with varying volumes of DOX and CUR working solutions. For the plasma samples, the final concentrations of DOX and CUR were 0.1–0.5 μg/mL and 0.05–0.4 μg/mL, respectively. For the urine samples, the concentrations were 0.2–0.5 μg/mL and 0.1–0.4 μg/mL, respectively. All tubes were mixed well and diluted with acetonitrile, a protein precipitating agent, to 10.0 mL. After that, each tube was subjected for 1 min to a vortex, and then it was centrifuged at a speed of 4000 rpm for 20 min. Subsequently, the clear supernatants were subjected to filtration utilizing 0.45 μm syringe filters. The next step was to sequentially transfer aliquots of 1 mL of the filtered supernatants into a series of 10.0 mL volumetric flasks, and after that, each flask was completed to the mark with ethanol. All synchronous spectrofluorimetric measurements were carried out along with blank plasma or urine samples and the diluting solvent was ethanol. The calibration graphs and regression equations were then derived.

## Results and discussion

**Fig. 2:**
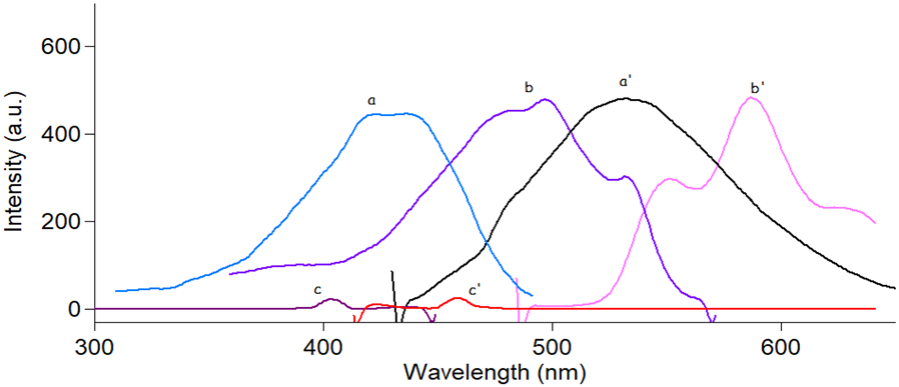
Excitation and emission fluorescence spectra of CUR (0.2 µg/mL) (**a**,** a′**) and DOX (0.5 µg/mL) (**b**, **b′**) in ethanol (**c**, **c′**)

**Fig. 3:**
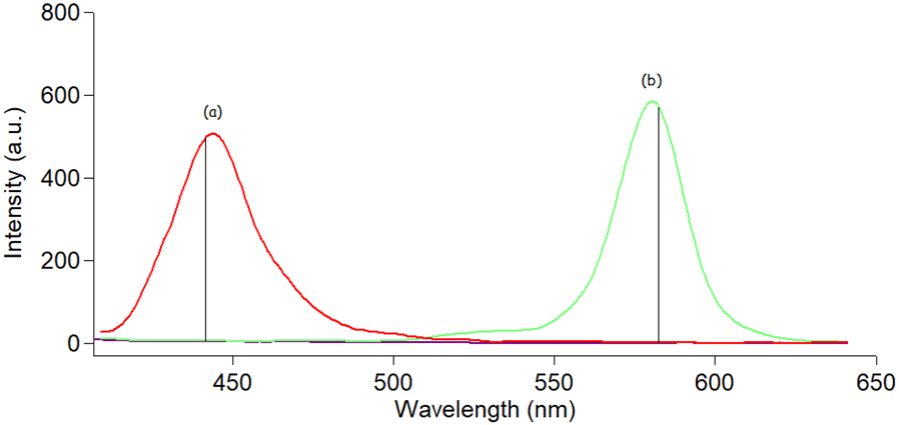
Synchronous fluorescence spectra of (**a**) CUR (0.4 µg/mL) and (**b**) DOX (0.3 µg/mL) in (**c**) ethanol (blank solvent)

**Fig. 4:**
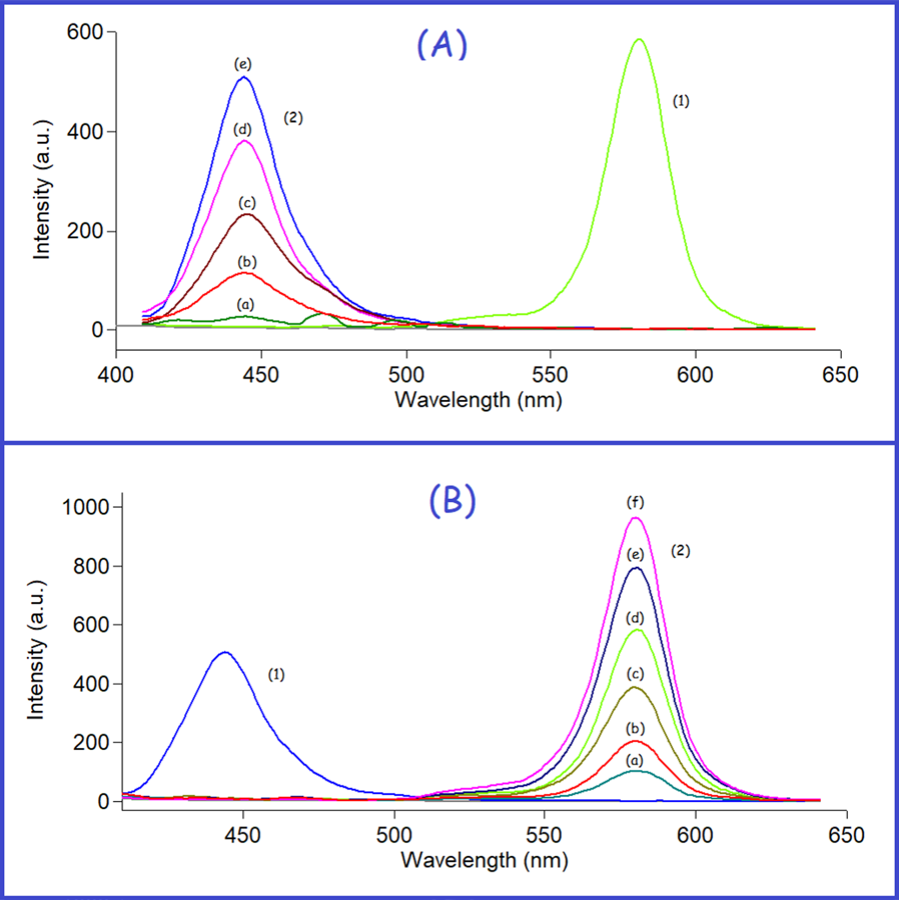
**A** Synchronous fluorescence spectra of (1) DOX (0.3 µg/mL) and (2) CUR (a–e: 0.04, 0.1, 0.2, 0.3, and 0.4 µg/mL) at 442.5 nm. **B** Synchronous fluorescence spectra of (1) CUR (0.4 µg/mL) and (2) DOX (a–f: 0.05, 0.1, 0.2, 0.3, 0.4, and 0.5 µg/mL) at 571.5 nm

Doxorubicin and curcumin showed strong native fluorescence at 595 and 535 nm after excitation at 495 nm and 420 nm for DOX and CUR, respectively, as abridged in Fig. 2. However, the emission spectra of CUR and DOX were highly overlapped which makes their simultaneous determination quite difficult. Consequently, the simultaneous measurement of both analytes in biological matrices via conventional fluorescence spectroscopy represents a significant challenge. Hence, the SFS approach was the best choice for analyzing these two analytes with high selectivity and minimal interference. Different Δλ in the range of (20–200 nm) were studied to choose the best optimum Δλ for the resolution of the studied mixture. It was noticed that Δλ = 20 nm yielded the optimal results in terms of getting resolved spectra for each analyte while avoiding any interference from the other one (Fig. 3). After using SFS method at Δλ = 20 nm, it was found that the SFS spectra of various concentrations of CUR were recorded at 442.5 nm in the presence of a fixed concentration of DOX (0.3 μg/mL) (Fig. 4A), while various concentrations of DOX were recorded at 571.5 nm in the presence of a fixed concentration of CUR (0.4 μg/mL) (Fig. 4B).

### The suggested approach optimization

Different parameters affecting the fluorescence intensity of both DOX and CUR were thoroughly examined. Each parameter is optimized separately, while the others are kept constant.

#### Effect of diluent

The impact of different diluents was investigated in order to choose the best one yielding the highest fluorescence intensity. Four solvents, including methanol, water, acetonitrile, and ethanol were examined.

**Fig. 5:**
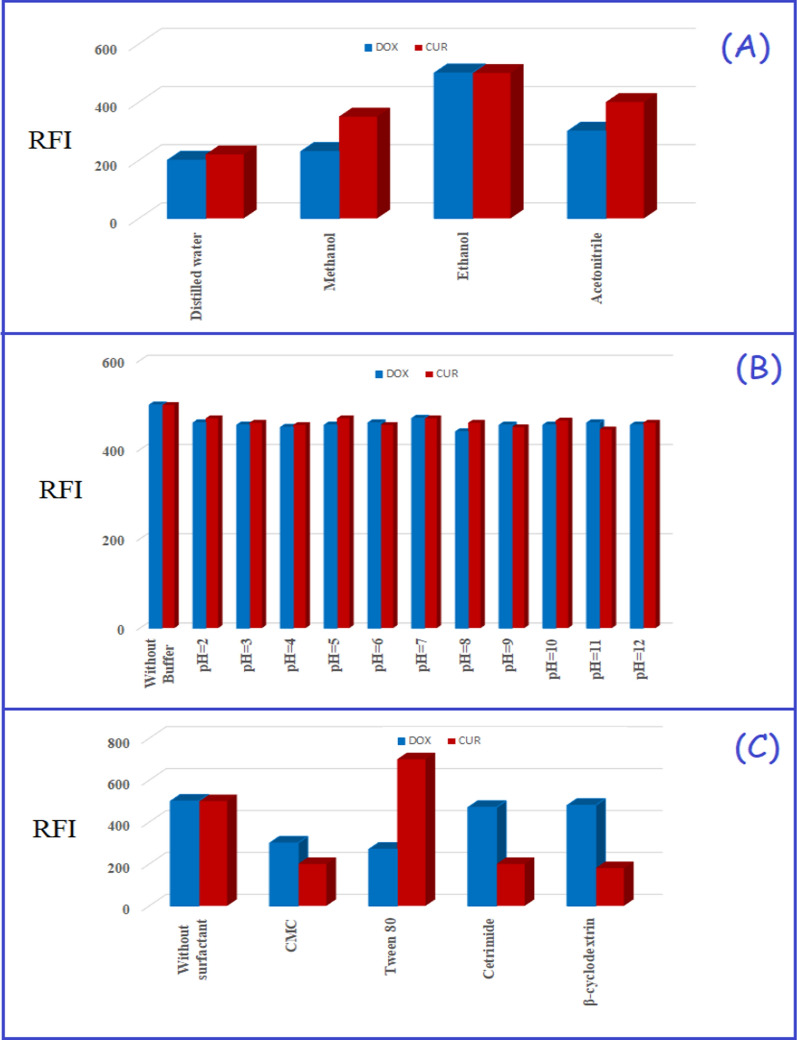
**A** The effect of diluting solvents. **B** The effect of pH. **C** The effect of surfactants and macromolecules

Using ethanol as the diluting solvent for both analytes exhibited the highest fluorescence intensities (Fig. 5A). Ethanol has the additional advantage of being a green and eco-friendly solvent so it is the best one of choice to be used for the suggested method.

#### Effect of pH of buffer solutions

The impact of pH on the fluorescence intensity of DOX and CUR was investigated utilizing BRB (pH range: 2–12). Neither DOX nor CUR showed a significant increase in fluorescence intensity in the studied range of pH as shown in Fig. 5B. Consequently, the present study was carried out without using any buffer solutions.

#### Effect of surfactants and macromolecules

The surfactant study was conducted to further enhance the sensitivity of the proposed method and reach lower limits of detection [51]. The impact of different surfactants (1.0% w/v) such as sodium dodecyl sulfate (SDS), Tween 80, carboxy methyl cellulose (CMC), or macromolecules such as β-cyclodextrin on the fluorescence intensity of DOX and CUR was carried out. It was observed that none of the specified surfactants or macromolecules significantly increased the fluorescence intensity of the two analytes as abridged in Fig. 5C. It can be explained that the bulkiness of the two analytes inhibits their inclusion in the micelles of organized media. Therefore, the proposed approach was carried out utilizing ethanol without any surfactants or macromolecules.

#### The optimum Δλ selection

Both DOX and CUR were recorded at Δλ intervals between 20 and 160 nm to select the best Δλ that gives peaks with high resolution and sensitivity. The ideal Δλ at which the highest selectivity and sensitivity were attained for both analytes was found to be Δλ = 20 nm (Fig. 3).

### Validation

The suggested approach was validated following the (ICH) Q_2_R_1_ guidelines for validation of analytical procedures [52].

Concerning the linearity of the suggested approach, it was linear throughout the final concentration range of 0.05–0.50 µg/mL and 0.04–0.40 µg/mL for DOX and CUR, respectively, and the regression data was abridged in Table 1. The linearity was established by the high values of the correlation coefficient (r > 0.999) for both drugs [53]. The sensitivity of the proposed approach was assessed by the calculation of limit of detection (LOD) and limit of quantitation (LOQ). The LOD and LOQ were calculated with the following equations in accordance with the (ICH) Q_2_R_1_ recommendations [52]:

$${\text{LOD}} = \frac{3.3 }{S},\quad {\text{LOQ}} = \frac{10 }{S},$$

where σ is the standard deviation of intercept and S is the slope.

**Table 1:** Analytical performance data for the proposed method

Parameter	DOX	CUR
Wavelength difference	$\Delta {\uplambda } =$ 20 nm	
Linearity range (μg/mL)	0.05–0.50	0.04–0.40
Intercept (*a*)	7.32	− 25.94
Slope (*b*)	1927.78	1339.42
Correlation coefficient (*r*)	0.9997	0.9998
S.D. of residuals (S_*y/x*_)	9.08	4.39
S.D. of intercept (S_*a*_)	7.07	3.69
S.D. of slope (S_*b*_)	23.30	15.06
Percentage relative standard deviation, % RSD	1.19	1.55
*%* Error	0.49	0.69
Limit of detection, LOD (µg/mL)	0.012	0.009
Limit of quantitation, LOQ (µg/mL)	0.037	0.028

The values of LOD were 0.012 and 0.009 µg/mL, while the values of LOQ were 0.037 and 0.028 µg/mL for DOX and CUR, respectively.

The accuracy of the suggested approach was assessed by performing a comparative analysis between the results obtained from the proposed approach and those of the reported approaches [16, 33]. The comparison method [16] for DOX involved the measurements of the native fluorescence of DOX at 590 nm after excitation at 475 nm using 0.5 mL of 0.5 M HCl as a diluting solvent and the volume was completed to the mark with ethanol. The comparison method [33] for CUR involved the measurements of the native fluorescence of CUR at 527 nm after excitation at 423 nm using methanol as a diluting solvent at a concentration range of 0.05–0.50 μg/mL. The statistical analysis results acquired through the utilization of Student’s t-test and variance ratio F-test showed no significant difference between the suggested approach and the reported approaches [53] as abridged in Table 2.

**Table 2:** Assay results for the determination of DOX and CUR in their pure forms by the proposed and comparison methods

Studied drugs	Proposed method			Comparison methods [16, 33]	
	Conc. taken (μg/mL)	Conc. found (μg/mL)	% Found^a^	Conc. taken (μg/mL)	% Found^a^
DOX	0.05	0.050	100.00	0.2	99.50
	0.1	0.101	101.00	0.5	100.40
	0.2	0.197	98.50	0.8	99.88
	0.3	0.299	99.67		
	0.4	0.407	101.75		
	0.5	0.496	99.20		
Mean ± S.D			100.02 ± 1.19	99.93 ± 0.45	
t-test			0.16 (2.37)*		
F-test			6.99 (19.30)*		
CUR	0.04	0.040	100.00	0.1	99.00
	0.1	0.102	102.00	0.2	101.00
	0.2	0.196	98.00	0.4	99.75
	0.3	0.304	101.33		
	0.4	0.399	99.75		
Mean ± S.D			100.22 ± 1.55	99.92 ± 1.01	
t-test			0.33 (2.45)*		
F-test			2.36 (19.25)*		

*The figures between parentheses are the tabulated t and F values at P = 0.05 [53]

^a^Each result is the average of three separate determinations

Intra-day and inter-day precision of the suggested approach were investigated for both DOX and CUR using three different concentrations three times within 1 day (intra-day precision) or within 3 different days (inter-day precision). High precision was confirmed by the relatively minimal values of percentage RSD (< 2.0) and percentage error (< 1.0) for both drugs (Table 3).

**Table 3:** Precision data for the determination of DOX and CUR pure forms by the proposed method

Conc (μg/mL)		DOX			CUR		
		0.1	0.3	0.5	0.1	0.3	0.4
Intraday	% Found^a^	100.30	100.57	99.83	100.67	99.23	100.22
		98.81	99.54	99.73	98.54	101.13	99.67
		100.89	100.00	100.46	100.14	99.64	99.94
	Mean	100.00	100.04	100.01	99.78	100.00	99.94
	S.D	1.07	0.51	0.39	1.11	1.00	0.28
	% RSD	1.07	0.51	0.39	1.11	1.00	0.28
	% Error	0.62	0.29	0.23	0.64	0.58	0.16
Interday	% Found	101.00	100.67	99.80	101.00	99.30	100.75
		102.00	99.67	99.20	102.00	101.33	99.70
		99.00	99.33	98.80	99.700	100.67	100.50
	Mean	100.67	99.89	99.27	100.90	100.43	100.32
	S.D	1.53	0.70	0.50	1.15	1.04	0.55
	% RSD	1.52	0.70	0.50	1.15	1.04	0.55
	% Error	0.88	0.40	0.29	0.66	0.60	0.32

^a^Each result is the average of three separate determinations

The robustness of the proposed approach was assessed by investigating the minor changes that could affect the fluorescence intensity such as Δλ = 20 ± 5 nm. These minor changes revealed no significant alteration in the proposed approach performance.

**Fig. 6:**
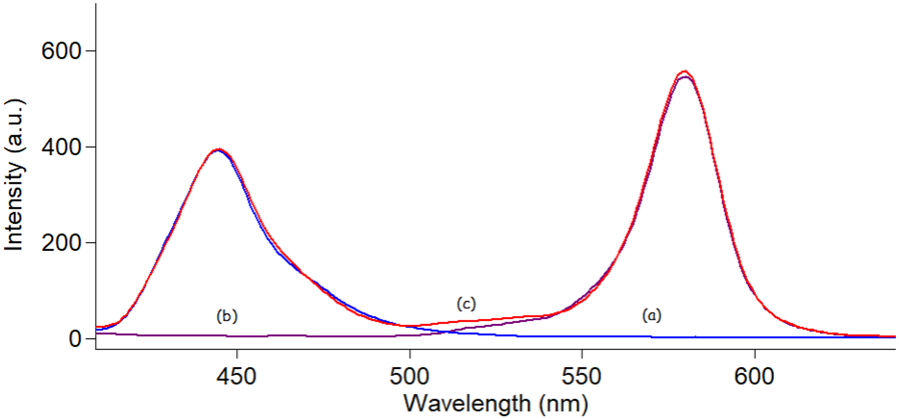
Synchronous fluorescence spectra of (**a**) CUR (0.3 µg/mL), (**b**) DOX (0.3 µg/mL), and (**c**) synthetic mixture of both

The selectivity was assessed for the proposed approach by the determination of DOX and CUR in various ratios of their laboratory-prepared mixtures, as shown in Fig. 6. At 571.5 nm, DOX was recorded without any CUR interference. At 442.5 nm, CUR was measured where DOX showed no interference. The selectivity was assessed by the excellent % recovery and low values of %RSD (< 2%) (Table 4). Additionally, the selectivity of the suggested approach was established by estimation of CUR and DOX in spiked human plasma and urine. It was found that the proposed approach showed low SD values for both analytes (Table 5).

**Table 4:** Assay results for the determination of DOX and CUR in synthetic mixtures by the proposed method

Mixture no.	Conc. taken (μg/mL)	Conc. taken (μg/mL)	Conc. found (μg/mL)	Conc. found (μg/mL)	% Found^a^	% Found^a^
	DOX	CUR	DOX	CUR	DOX	CUR
1	0.4	0.2	0.407	0.203	101.97	101.83
2	0.1	0.3	0.098	0.302	98.15	101.14
3	0.2	0.4	0.203	0.406	101.69	100.71
4	0.4	0.3	0.399	0.303	99.78	101.06
Mean					100.40	101.19
± S.D					0.79	0.47
% RSD					0.79	0.47

The figures between parentheses are the tabulated t and F values at P = 0.05 [53]

^a^Each result is the average of three separate determinations

**Table 5:** Assay results for the determination of DOX and CUR in spiked human plasma and urine samples

Parameters	DOX			CUR		
	Conc. taken (μg/mL)	Conc. found (μg/mL)	% Found^a^	Conc. taken (μg/mL)	Conc. found (μg/mL)	% Found^a^
Human plasma	0.1	0.098	98.00	0.05	0.049	98.00
	0.2	0.198	99.00	0.1	0.103	103.00
	0.4	0.409	102.25	0.3	0.295	98.33
	0.5	0.494	98.80	0.4	0.403	100.75
Mean			99.51			100.02
± S.D			1.88			2.34
r			0.9995			0.9997
Regression equation			RSFI = 442.68c − 23.52			RSFI = 722.9c − 9.37
Urine	0.2	0.199	99.5	0.1	0.099	99.00
	0.4	0.407	101.75	0.3	0.308	102.67
	0.5	0.494	98.8	0.4	0.394	98.50
Mean			100.02			100.06
± S.D			1.54			2.28
r			0.991			0.996
Regression equation			RSFI = 99.29c + 91.93			RSFI = 206.79c + 53.36

^a^Each result is the average of three separate determinations

## Applications

### Assay of DOX/CUR in laboratory-prepared mixtures

The adopted approach allowed the simultaneous estimation of CUR and DOX in their laboratory-prepared mixtures with various concentration ratios as abridged in Fig. 6. The percentage recoveries for each drug were then calculated from the regression equation for each drug. As cited in Table 4, the results confirmed the accuracy of the suggested approach.

### Assay of DOX/CUR in plasma and urine samples

The adopted approach exhibits sufficient sensitivity and selectivity that enable the simultaneous quantification of CUR and DOX in human plasma or urine samples because their maximum plasma concentration (C_max_) falls within the linearity range of the suggested approach [54, 55]. As cited in Table 5, there was a linear correlation obtained when synchronous fluorescence intensity was plotted versus the concentrations of each drug in μg/mL for spiked plasma or urine matrices. The suggested method achieved high % recoveries and minimal % RSD values. These findings confirmed the high efficiency of the suggested approach in such complicated matrices as shown in Table 5.

### Greenness assessment

Owing to the numerous solvents and chemicals used throughout analytical procedures, the environment is significantly influenced; as a result, it is of utmost importance to protect the environment from the waste produced by such procedures. Different three metrics were applied in this study to assess the proposed approach greenness: National Environmental Method Index (NEMI), analytical ecoscale, and AGREE evaluation method.

NEMI [48] is considered a qualitative approach, that evaluates the environmental effect of analytical procedures by employing a pictogram. This pictogram is divided into four portions. Each portion was colored green if (I) the utilized reagents not regarded to be persistent, bioaccumulative or toxic, (II) the utilized reagents are not on hazardous waste list, (III) the pH used in the proposed approach not less than 2.0 or more than 12, (IV) the waste amount generated by the suggested approach was less than 50 g per sample. Consequently, the developed method met the four criteria of greenness of NEMI tool, as abridged in Table 6.

**Table 6:** Results for evaluation of greenness of the proposed methods

1. National Environmental Method Index (NEMI) pictogram [48]			

2. Analytical eco-scale score [49]			

Item	No of pictogram	Word sign	Penalty points
(1) Reagent; volume (mL)			
Ethanol < 10 mL	2	Danger	4
(2) Spectrofluorimeter; < 0.1 KWh per sample			0
(3) Occupational hazard			0
(4) Waste			3
Total penalty points			7
Analytical eco-scale score			93
3. AGREE assessment [50]			

Analytical eco-scale [49] is a semiquantitative tool used to evaluate the overall environmental impact of an analytical procedure. The calculation of the total penalty points in each stage is conducted by considering factors such as hazards, instrumentation energy, reagent amount, and waste. Following that, the sum of penalty points was subtracted from a value of 100, yielding the analytical eco-scale value. As shown in Table 6, the proposed approach’s score was 93, indicating the method’s excellent greenness.

AGREE [50], is a tool for assessing the proposed methods’ greenness by assessing important 12 principles. The outcome from the AGRRE pictogram indicates a score from 0 to 1. Table 6 shows that the suggested approach has a high score and satisfactory results, indicating an ‘excellent green’ method.

## Conclusion

The current study aimed to propose a green synchronous spectrofluorimetric approach for the simultaneous estimation of curcumin and doxorubicin. The advantages of the suggested approach include selectivity, sensitivity, reliability, and precision. Moreover, compared with chromatographic approaches, the suggested procedure requires a short time without the need for complicated sample treatment steps. The method was optimized and validated to allow the simultaneous estimation of CUR and DOX in pure form and spiked human plasma and urine. The sensitivity of the suggested approach is superior down to 0.028 and 0.037 µg/mL for CUR and DOX, respectively, so it is considered a good choice for therapeutic drug monitoring of DOX and CUR. Furthermore, the suggested method has a low environmental effect due to the usage of a green solvent, ethanol, which is safe and eco-friendly.

## Data Availability

The datasets generated during and/or analysed during the current study are available in the Dryad repository: https://datadryad.org/stash/share/qVgAz5opoxEXBEGNKXP9ar_a0x9ZgCP1N4b7z7hmrAY.
